# Inflammatory pseudotumor of the liver: a case report and review of the literature

**DOI:** 10.1186/1752-1947-5-196

**Published:** 2011-05-21

**Authors:** Achilleas Ntinas, Dimitrios Kardassis, Dimosthenis Miliaras, Konstantinos Tsinoglou, Athanasios Dimitriades, Dionisios Vrochides

**Affiliations:** 1 Department of Hepato-Pancreato-Biliary Surgery, Euromedica General Clinic, 2 Gravias - 11 Maria Callas Str, 546 45, Thessaloniki, Greece; 2 Laboratory of Histology and Embryology, Medical School, Aristotle University, 54006, Thessaloniki, Greece

## Abstract

**Introduction:**

Inflammatory pseudotumor of the liver represents a fairly uncommon pathology. Although it is a benign tumor, the correct diagnosis can be missed.

**Case presentation:**

We report the case of a 55-year-old Caucasian man, who presented with a one-month history of abdominal pain and weight loss. He was diagnosed with a primary liver tumor by computed tomography and magnetic resonance imaging. Alpha-fetoprotein levels ranged within normal limits. A right posterior sectorectomy was performed. Histopathology revealed an inflammatory pseudotumor of the liver. Our patient remains in good condition one year later.

**Conclusion:**

Although inflammatory pseudotumor of the liver is usually a benign process, controversy regarding its management still exists. With this case report we review the existing literature and consider hepatectomy as a safe treatment approach.

## Introduction

Inflammatory pseudotumors (IPTs) are reactive conditions that occur in many organs. They have been reported in the lung, which is the most common site of occurrence, central nervous system, major salivary glands, kidney, liver, omentum, ovary, larynx, urinary bladder, breast, pancreas, spleen, lymph nodes, skin, soft tissues, orbit etc. [1]. The first report of an hepatic IPT was published by Pack and Baker in 1953 [2]. This entity has also been described as inflammatory myofibroblastic tumor, plasma cell granuloma, histiocytoma and fibroxanthoma. Someren classified inflammatory pseudotumors into three groups according to histology. The first of these is xanthogranuloma-type pseudotumors, the second is plasma cell granuloma-type pseudotumors and finally there are sclerosing pseudotumors [3]. Macroscopically, the lesion may mimic a malignancy and may be single or multiple. Its dimensions may be up to 25 cm. Microscopically, IPT is characterized by spindle-shaped cells, myofibroblasts and mixed inflammatory cells (plasma cells, lymphocytes and, sporadically, histiocytes).

The literature survey to date indicates 130 publications with 289 cases of hepatic IPTs [4-6]. The prevalence of this condition, although low, is not irrelevant. In a retrospective analysis of 403 patients who underwent surgery for focal liver lesions, the incidence of IPTs was 0.7% [6]. Published data report that out of 188 patients with IPT, 106 underwent liver resection and 76 were treated conservatively, 5 out of whom subsequently also had to undergo surgery because their initial medical treatment failed [4]. Upon diagnosis, the proposed treatment of IPTs is rather conservative, administering either antibiotics or steroids [7]. However, occasional reports advocated the use of more radical treatment, that is to say, its resection [1,4].

## Case presentation

**Figure 1:**
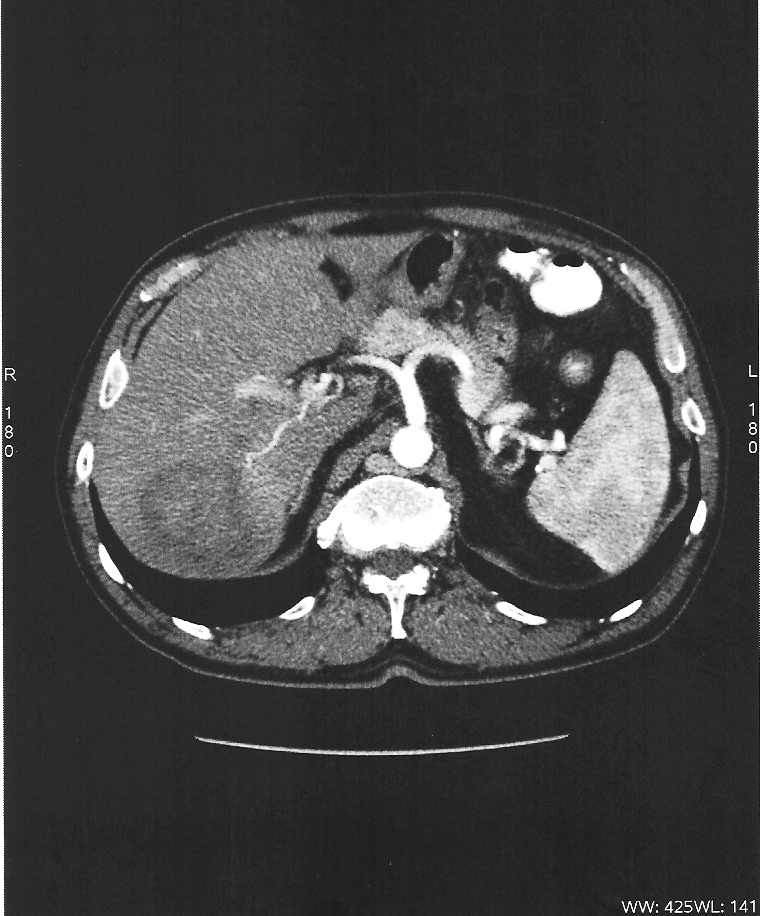
**CT scan showing a well-encapsulated round tumor, with low density and low enhancement of the tumor during the arterial phase**.

We report on a 55-year-old Caucasian man, who presented with a three-day history of right upper quadrant abdominal pain. He also complained of anorexia and weight loss (5 kg) in one month. He had no fever or night sweats and he presented without jaundice. Our patient was submitted to a chest X-ray, ultrasonography (USG) of the abdomen, triple-phase contrast computed tomography (CT) and magnetic resonance imaging (MRI) of the abdomen. As part of the metastatic work-up he was also submitted to a chest CT, esophagogastroscopy and colonoscopy. A laboratory blood test for serum albumin, serum total bilirubin, alanine aminotransferase, aspartate aminotransferase, prothrombin time, hepatitis B surface antigen, and antibodies to hepatitis C, serum alpha-fetoprotein (AFP), carcinoembryonic antigen and carbohydrate antigen 19-9 (CA 19-9) were obtained. An abdominal CT and MRI showed a well-defined heterogeneous mass situated in his right hepatic lobe measuring 4.0 cm × 4.0 cm (Figure 1). The lesion featured central necrosis, a hyper-dense rim and a mild enrichment from the arterial phase in the CT, thus conformable to malignancy. A metastatic work-up was negative. Laboratory investigations showed normal liver function tests, no hepatitis B or C infection, no leucocytosis and normal AFP and CA 19-9 levels. The diagnosis of primary hepatic malignant tumor and the indication of its resection were strongly suspected by the tumor board of our institute. A right posterior sectorectomy was performed with the aid of intra-operative USG. The lesion was found in Couinaud's segments 6 and 7. The right sub-diaphramatic space was drained for 72 hours and intra-operative blood loss was 180 ml. The post-operative course was uneventful. Our patient was discharged on the eighth post-operative day.

**Figure 2:**
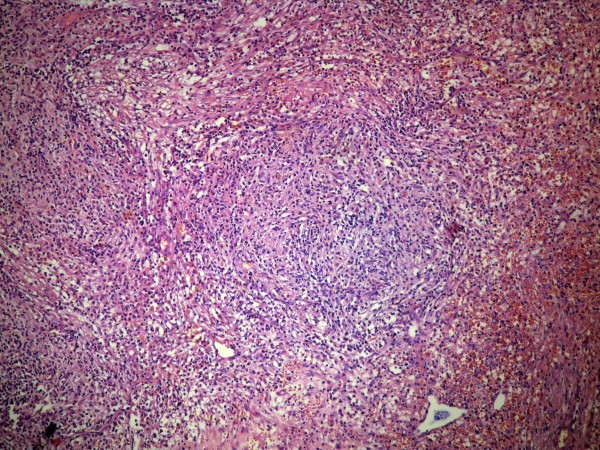
**Macrophages are forming aggregates, while the rest of the inflammatory cells are interspersed in a stroma with abundant fibroblasts and collagen bundles**. The inflammation partially extends to the adjacent liver parenchyma. The aggregate of macrophages is located in the lower right part of the image (hematoxylin and eosin, × 400).

**Figure 3:**
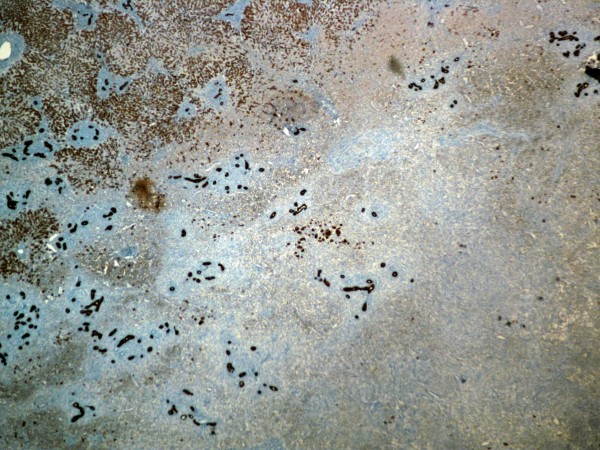
**The cells of the lesion are negative to broad-spectrum keratin antibody**. In contrast, the normal liver parenchyma on the left upper part of the image, and many bile ducts, which are confound into the tumor, are positive to keratin (DAB, × 25).

The specimen of liver parenchyma that was delivered for histopathological evaluation was 254 g in weight. It measured 12.5 cm × 11.0 cm × 5.5 cm. A white/yellow, relatively well-circumscribed lesion, 4.0 cm in diameter, was identified. The removal of the tumor was complete. Microscopic examination revealed a process with benign characteristics, which included numerous inflammatory cells - mature plasma cells, lymphocytes, eosinophils and macrophages, most of them with xanthomatous changes (Figure 2). To exclude the possibility of carcinoma and dendritic sarcoma, monoclonal antibodies against broad-spectrum keratin (AE1/AE3, Dako, Glostrup, Denmark), and CD21 (Dako) were applied through a standardized immunohistochemical method (Nexes, Ventana, Tuscon, USA). Both antibodies had negative reactions in the cells of the lesion (Figure 3). In addition, many of the fibroblasts presented myofibroblastic phenotype, since they were positive to an antibody against α-smooth muscle actin (Novocastra, Newcastle, UK). A well differentiated hepato-carcinoma was ruled-out and the final diagnosis of hepatic IPT was made. Our patient received no further treatment. He remains in good condition one year later.

## Discussion

The etiology and pathogenesis of IPTs remain unknown [4]. Frequently an infectious agent is implicated. In many reports, the responsible microorganisms found include *Bacteroides caccae*, *Actinomyces*, *Klebsiella*, *Escherichia coli*, Gram-positive cocci and β-hemolytic *streptococcus *[7]. This could explain why, in some cases, the decrease or even resolution of IPTs occurs after treatment with antibiotics. However, in other reports, no causative microorganisms have been identified, which could imply the association of IPTs with hepato-pancreato-biliary autoimmune diseases, such as IgG4 sclerosing cholangitis [8]. Based on this, many reports suggest therapeutic management (tumor resolution) with steroid administration. Differentiating between IPTs and other focal hepatic lesions remains a major problem, despite progress in imaging technology. Unfortunately, IPTs may mimic malignant lesions (lymphoma, malignant fibrous histiocytoma, hepatocellular carcinoma, metastatic tumor, etc.) and granulomatous lesions (tuberculosis and sarcoidosis) [4]. The CT-scan usually reveals lesions with variable contrast enhancement. IPTs may present with hypovascular character because of fibrosis and also show a delayed enhancement, similar to metastatic liver tumors and cholangio-carcinomas [7]. The MRI may produce low signal intensity (hypointense) on T1-weighted images with moderate to high signal intensity (hyperintense) on T2 sequence [4,7]. Because MRI provides additional information about hepatic lesion characteristics, it is often used in our institute in the metastatic work-up.

In general, it is difficult to distinguish IPTs from malignant tumors by radiographic studies. Literature shows that in some (individual) cases a percutaneous tumor biopsy provided the correct diagnosis. These patients were treated with antibiotics and/or corticosteroids with complete resolution of the lesion; however, some of these lesions have recurred [9]. In contrast, there have been numerous studies reported where hepatic resection was employed, mainly due to the pre-operative malignant radiographic appearance of the tumor, and incidentally resected IPTs never recurred [10].

A further problem suggested in the literature, besides recurrence, is the potential malignant transformation of IPTs some years after hepatectomy. It has even been reported that some patients died of this disease. In particular, the authors report that these patients developed non-Hodgkin lymphoma, hepatic sarcoma and recurrence of IPTs with multiple hepatic masses and extension to the thorax some years later [11,12].

Regarding the current case, liver biopsy was not used as a diagnostic tool due to the strong suspicion of the lesion's malignant character, provided by the imaging findings. While liver biopsy has an undoubted role in the investigation and management of liver metastases of unknown origin, it plays a doubtful and probably dangerous role in patients with a solitary hepatic mass with high probability of malignancy [13]. Needle tract seeding has been reported to occur in 5.1% of patients with hepatocellular carcinoma (HCC), who underwent percutaneous needle biopsy [14]. Hemorrhage, with mortality rates less than or equal to 1 in 10,000 biopsies in experienced centers, is another possible, although rare, complication [13]. Furthermore, for the planning of a liver surgical intervention, biopsy of the tumor is not necessary.

## Conclusion

A fairly uncommon clinical entity was presented. IPTs are often difficult to distinguish from hepatic malignant tumors. The safety of hepatectomy of non-cirrhotic patients has increased over the past 20 years, with mortality converging to 0%, therefore surgical resection should be considered as treatment of choice in such cases [15]. This approach is preferable because it not only minimizes the risk of a biopsy-related complication (dissemination in cases of malignancy), but it also abolishes the possibility of IPT recurrence.

## Consent

Written informed consent was obtained from the patient for publication of this case report and any accompanying images. A copy of the written consent is available for review by the Editor-in-Chief of this journal.

## Competing interests

The authors declare that they have no competing interests.

## Authors' contributions

AN wrote the manuscript. DK reviewed the literature and corrected the manuscript. DM performed the pathology tests of the specimen. KT interpreted the CT and MRI. AD corrected the manuscript and DV conceived the idea and corrected the manuscript. All authors have read and approved the final manuscript.
